# Subspecies distribution and drug-resistance characteristics of *Mycobacterium abscessus* complex clinical isolates in South China

**DOI:** 10.1128/spectrum.04103-23

**Published:** 2025-03-19

**Authors:** Xinyu Wang, Biyi Su, Pinru Chen, Haobin Kuang, Ping Guan, Chao Zhang, Liping Pan, Jialou Zhu, Yaoju Tan

**Affiliations:** 1 State Key Laboratory of Respiratory Disease, Guangzhou Key Laboratory of Tuberculosis Research, Department of Clinical Laboratory, Guangzhou Chest Hospital, Institute of Tuberculosis, Guangzhou Medical University, Guangzhou, Guangdong, China; 2 Beijing Key Laboratory for Drug Resistant Tuberculosis Research, Beijing Chest Hospital, Capital Medical University, Beijing Tuberculosis and Thoracic Tumor Research Institute, Beijing, China; 3 State Key Laboratory of Respiratory Disease, Guangzhou Key Laboratory of Tuberculosis Research, Department of Tuberculosis, Guangzhou Chest Hospital, Institute of Tuberculosis, Guangzhou Medical University, Guangzhou, Guangdong, China; Shenzhen University School of Medicine, Shenzhen, China

**Keywords:** *Mycobacterium abscessus*, whole-genome sequencing, mutation, drug resistance, single-nucleotide polymorphism

## Abstract

*Mycobacterium abscessus* complex (MABC) is the most common rapidly growing non-tuberculous mycobacterium (NTM) and a significant pathogen responsible for various infections. Subspecies of MABC exhibit differing levels of antibiotic resistance, potentially influencing patient prognosis. In this study, 196 MABC clinical isolates were collected from Guangzhou Chest Hospital and subjected to a drug sensitivity test (DST) followed by whole-genome sequencing. In our study, the most common subspecies was *Mycobacterium abscessus* subsp. *abscessus*, followed by *M. abscessus* subsp. *massiliense*, with *M. abscessus* subsp. *bolletii* being the least frequent. The isolates were most susceptible to tigecycline and amikacin, followed by clarithromycin and linezolid. Bedaquiline and clofazimine showed promising anti-bacterial activity, whereas doramapimod and capreomycin demonstrated limited efficacy. The *erm(41*) gene mutation at position 28 and the *rrl* gene at position 2270 were identified in clarithromycin-resistant isolates. Additionally, the *rrs* gene mutation at position 1375 may serve as an indicator of amikacin resistance. Regular molecular surveillance investigation of NTM and DST is recommended for improved management.

**IMPORTANCE:**

As Guangdong is a region with a high prevalence of NTM, this study investigates the epidemiological trends of MABC subspecies and their associated drug-resistance profiles, addressing a critical research gap. The findings are crucial for guiding hospital drug management and clinical decision making, enabling more accurate diagnoses and the formulation of personalized, effective treatment plans. Additionally, this study provides a valuable reference for the development of drug-resistance detection reagents and new anti-bacterial agents.

## INTRODUCTION

Non-tuberculous mycobacterium (NTM) is an environmental bacterium associated with chronic lung diseases, and its global prevalence has been increasing (1). In China, the rate of NTM isolation rose from 4.3% in 1979 to 11.1% in 2000, and further to 22.9% in 2010 (2). Between 2017 and 2022, there was a significant increase in the number of samples tested for MTB and/or NTM each year, accompanied by a notable annual rise in NTM detection (3). According to the national incidence survey of NTM conducted in China in 2021, 6.4% (317 of 4,917; 95% confidence interval 5.8%-7.2%) were confirmed to have NTM infection (4). Notably, the NTM isolation rate in the Guangzhou region exceeded the national average, with *Mycobacterium abscessus* complex (MABC) accounting for 23.1% of cases—second only to *Mycobacterium avium* complex (52.6%)—as a causative agent of NTM infections in Guangzhou and other parts of China (5). MABC, a rapidly growing subtype of NTM, exhibits multidrug resistance and is increasingly associated with infections in individuals with underlying structural lung conditions, such as cystic fibrosis (CF), non-CF bronchiectasis, chronic obstructive pulmonary disease, and those who are immunocompromised (6). MABC comprises three distinct subspecies: *M. abscessus* subsp. *abscessus* (Mab), *M. abscessus* subsp. *massiliense* (Mma), and *M. abscessus* subsp. *bolletii* (Mbo) (7). However, the differentiation of these subspecies is seldom considered in clinical diagnosis and treatment in China.

The clinical management of MABC presents distinct challenges due to the limited number of effective antibiotics, prolonged treatment durations, and frequent occurrence of treatment-related toxicities (8). Treatment typically involves a multidrug regimen that includes an oral macrolide (azithromycin or clarithromycin) and intravenous aminoglycoside (amikacin), combined with intravenous β-lactams like imipenem or cefoxitin (9, 10). However, treatment outcomes are often unsatisfactory, with relapse occurring even after prolonged therapy(18 months or more) (11). Improved understanding of enhanced insight into subspecies identification and isolate characteristics could help predict treatment outcomes in patients with MABC lung disease (12, 13). Notably, treatment efficacy may be influenced by macrolide resistance, as shown in a study where sputum conversion was observed in 88% of patients with clarithromycin-susceptible Mma, compared to just 25% in those with clarithromycin-resistant Mab (13). Furthermore, acquired drug resistance to amikacin and macrolides is associated with mutations in the ribosomal RNA genes *rrs* (16 S rRNA) and *rrl* (23 S rRNA), respectively (14). Additionally, upregulation of the erythromycin ribosome methyltransferase [*erm(41)*] gene leads to inducible resistance to macrolides (15). Mab strains carry a fully functional *erm(41*) gene, which results in inducible macrolide resistance (16, 17). In contrast, most Mma strains lack inducible resistance due to a non-functional *erm(41*) gene with a 271 bp gap (18). This discrepancy may explain the higher treatment response rates observed in patients with Mma lung disease when treated with clarithromycin-based combination therapy, compared to those with Mab lung disease (13).

This study aims to investigate the distribution of MABC subtypes in clinical isolates from Guangzhou Chest Hospital. It compares the drug-resistance profiles of various MABC subtypes and phenotypes and examines the correlation between *in vitro* drug susceptibility to clarithromycin and amikacin and the associated genetic mutations.

## RESULTS

### Prevalence of MABC subspecies in Guangdong

**Fig 1:**
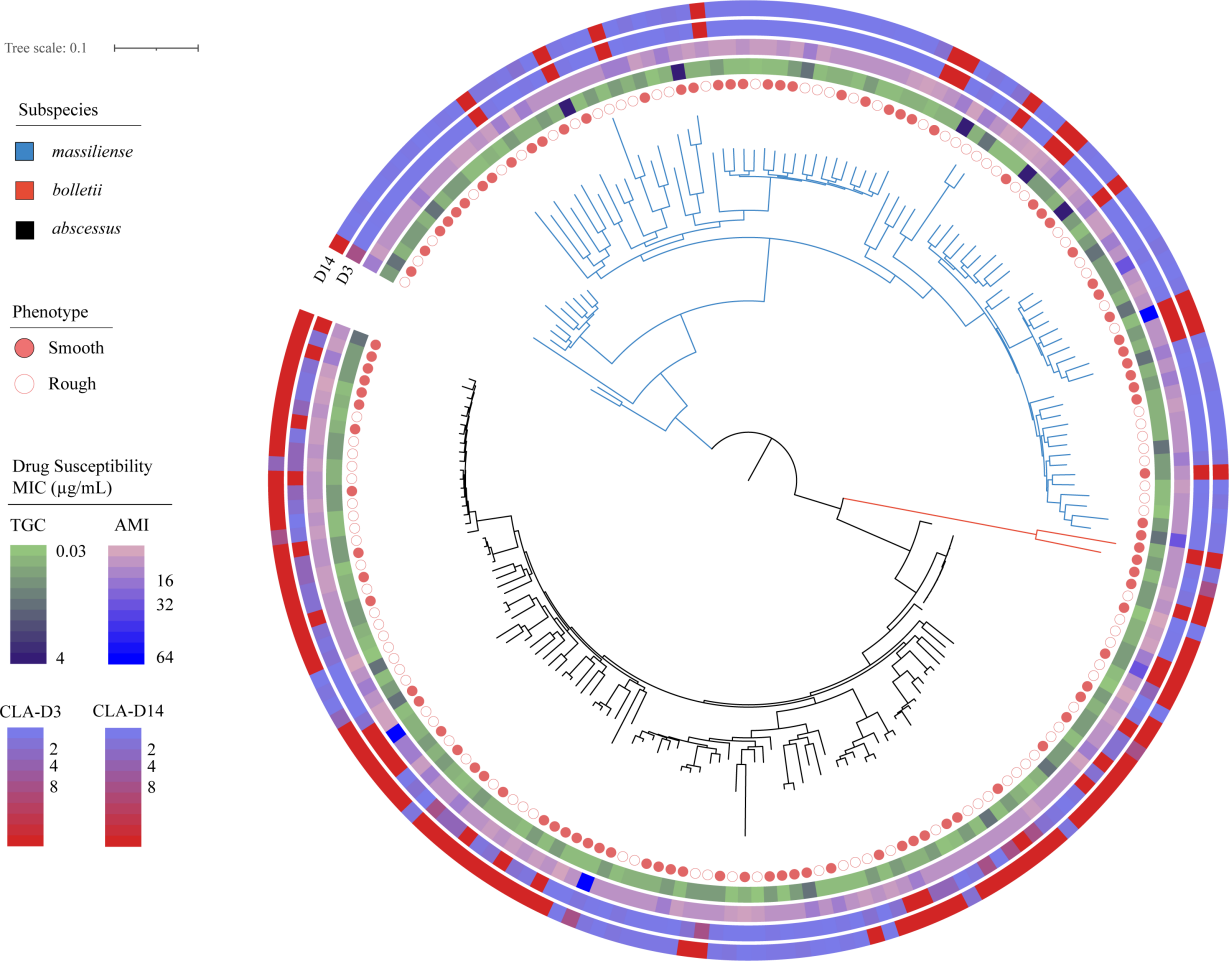
Phylogenetic tree of clinical isolates of *Mycobacterium abscessus* complex. Maximum likelihood phylogenetic tree of 196 *M*. *abscessus* isolates generated by IQ-TREE and displayed by iToL with corresponding subspecies and phenotypic groups, annotated for drug-resistance phenotypes (tigecycline, amikacin, and clarithromycin minimum inhibitory concentration [MIC], days 3 and 14).

Among the 196 clinical MABC isolates, Mab was the most prevalent (52.6%, 103 of 196), followed by Mma (45.4%, 89 of 196), and Mbo was the least common (1.0%, 2 of 196). Interestingly, two strains exhibited characteristics of both Mab and Mma (1.0%, 2 of 196). The resulting phylogenetic tree is shown in Fig. 1. To investigate potential patient-to-patient transmission in this hospital, we performed whole-genome single-nucleotide polymorphism (SNP) analysis and compared SNP differences among the isolates. Previous studies have shown differences of up to seven SNPs between environmental and clinical isolates (19). In this study, we applied the same threshold of seven SNPs. No isolates with ≤7 SNPs were identified, indicating no evidence of person-to-person transmission among the isolates.

### Characteristics and colony morphology of MABC

A total of 196 MABC strains were collected from 195 patients, 124 of whom were long-term residents of Guangdong province. Half of these residents (62 of 124, 50.0%) were from Guangzhou city (Fig. S1). Among the 196 MABC strains, approximately half exhibited rough (R) morphology (98 of 196, 50%), while the other half displayed smooth (S) morphology (Table S2). Importantly, no statistically significant difference was observed in the distribution of smooth and rough colony morphologies across different subspecies (*χ*^2^ = 1.832, *P* = 0.483).

### Sociodemographic data of patients

Due to limited number of patients with Mbo isolates, this study primarily compares clinical data from 103 Mab patients and 89 Mma patients. Various parameters were examined, including total patient count, hospitalization rates, average hospital stay duration, gender distribution, mean age, MABC culture instances, and clinical diagnoses (Table 1).

**TABLE 1:** Clinical information for patients with *Mycobacterium abscessus* complex^*b*^

	Mab	Mma	*P*
Total number of isolates (*n*)	103	89	
Total number of patients (*n*)	102^*a*^	89	
Number of inpatients (*n*)	26	15	
Average length of hospitalization (days) (*n* [IQR])	16 (13–26)	16 (14–22)	0.811
Sex (*n*)		71	
Female	70	40	0.001
Male	32	49	
Average age (years) (*n* [IQR])	49 (46–52)	48 (44–51)	0.318
Number of cultures in our hospital (*n*)			
Only once	29	43	0.005
More than once	73	46	
Diagnoses (*n*)			
NTM lung disease	55	37	0.019
NTM lung disease with TB	11	10	0.921
NTM lung disease with old TB	12	5	0.137
Pulmonary TB	15	20	0.166
Old TB	2	10	0.008
Other diseases of the lungs	5	4	1.000
Other	3	3	1.000
Other diagnoses (*n*)			
Bronchiectasis	64	38	0.006
Extrapulmonary tuberculosis	6	5	0.938
Hepatitis	13	5	0.093
Pneumonia	5	4	1.000
Diabetes	4	4	1.000
COPD	2	2	1.000
Hypertension	4	1	0.451

^*a*^ Two isolates collected from a single patient.

^*b*^ COPD, chronic obstructive pulmonary disease; Mab, *Mycobacterium abscessus* subsp. *abscessus*; Mma, *Mycobacterium abscessus* subsp. *massiliense*; NTM, non-tuberculous mycobacterium; TB, tuberculosis.

Among Mab patients, 76.5% (78 of 102) were diagnosed with NTM pulmonary disease, among whom 14.1% (11 of 78) had concurrent pulmonary tuberculosis, and 15.4% (12 of 78) had a history of previous tuberculosis. Notably, one patient had NTM skin infection. Among the Mma patients, 58.4% (52 of 89) were diagnosed with NTM pulmonary disease, including 19.2% (10 of 52) with coexisting pulmonary tuberculosis and 9.6% (5 of 52) with a history of prior tuberculosis. Furthermore, a significantly higher proportion of female patients was observed in the Mab group compared to the Mma group (*P* = 0.001). The Mab group also had a significantly higher prevalence of multiple isolations of the same strain in the hospital (*P* = 0.005). Additionally, bronchiectasis was more prevalent among Mab cases, with 62.1% (64 of 103) of patients, compared to 42.7% (38 of 89) in the Mma group, indicating a significant difference between the two subgroups (*P* = 0.006).

### Clinical relevance and sample collection time of MABC

**Fig 2:**
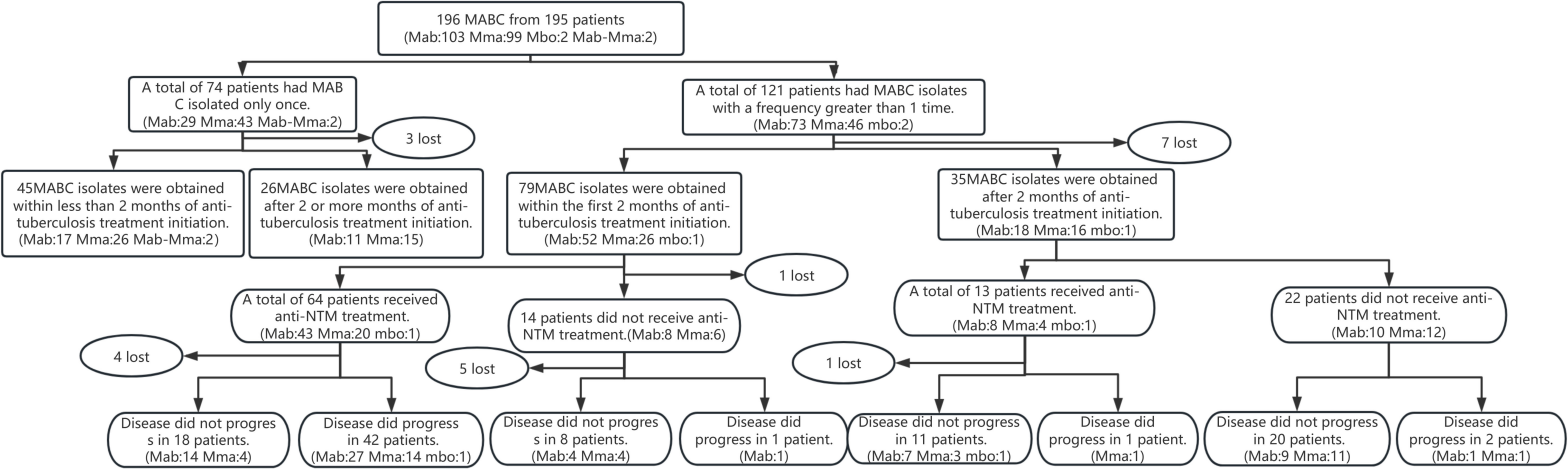
Relationship between time of MABC isolation, duration of anti-tuberculosis treatment, and progression of pulmonary disease. Mab, *Mycobacterium abscessus* subsp. *abscessus*; MABC, *Mycobacterium abscessus* complex; Mma: *Mycobacterium abscessus* subsp. *massiliense*; Mbo: *Mycobacterium abscessus* subsp. *bolletii*.

Out of 195 patients, 74 patients had a single instance of MABC isolation during their hospital stay, while 121 patients had recurrent MABC isolation, aligning with the primary diagnostic criteria. Within this recurring group, 35 patients had MABC isolated after 2 months of anti-tuberculosis treatment. Of these, 13 patients underwent extended anti-NTM treatment exceeding the 2 month mark. Notably, apart from 1 patient with concurrent drug-resistant tuberculosis, 11 patients showed no signs of disease progression, and 1 patient’s status was lost due to discontinued follow-up. Furthermore, 22 patients in this group deviated from standard anti-NTM treatment. Of these, 20 patients experienced no disease progression, while 2 patients showed disease advancement alongside concurrent pulmonary tuberculosis infection. Further details are presented in Fig. 2.

### Drug sensitivity test results for MABC

Out of 196 total isolates, drug sensitivity test (DST) was performed on 191 clinical MABC isolates, including 100 Mab strains, 89 Mma strains, and 2 Mbo strains. A detailed comparison of drug susceptibility outcomes between Mab and Mma strains is provided in Table 2.

**TABLE 2:** Comparison of drug sensitivity between Mab and Mma subspecies

Drugs^*a*^	Subspecies	MIC50 (µg/mL)	MIC90 (µg/mL)	R^*b*^ (*n*)	I^*b*^ (*n*)	S^*b*^ (*n*)	*χ* ^2^	*P*
TGC	Mab	0.5	1	0	–^*g*^	100	3.795	0.051^*d*^
	Mma	0.5	2	5	–	84		
AMI	Mab	8	16	2	1	97	0.851	0.842^*f*^
	Mma	8	16	1	2	86		
CLA	Mab	2	>16	27	11	62	14.898	0.000^*c*^
	Mma	0.12	>16	15	0	74		
LZD	Mab	32	>32	50	21	29	0.763	0.715^*c*^
	Mma	16	32	39	22	28		
DOX	Mab	>16	>16	97	1	2	4.920	0.177^*f*^
	Mma	>16	>16	82	6	1		
SXT	Mab	>8/152	>8/152	98	–	2	NA	0.499^*e*^
	Mma	>8/152	>8/152	89	–	0		
MIN	Mab	>8	>8	98	1	1	23.324	0.000^*c*^
	Mma	>8	>8	66	11	12		
FOX	Mab	128	>128	71	28	1	2.099	0.275^*f*^
	Mma	128	>128	57	32	0		
MXF	Mab	>8	>8	98	2	0	3.631	0.116^*f*^
	Mma	>8	>8	82	5	2		
TOB	Mab	16	>16	96	4	0	0.602	0.438^*d*^
	Mma	16	>16	88	1	0		
IMI	Mab	>64	>64	98	2	0	0.000	1.000^*d*^
	Mma	>64	>64	88	1	0		
CIP	Mab	>4	>4	100	0	0	NA	
	Mma	>4	>4	89	0	0		
FEP	Mab	>32	>32	100	0	0	NA	
	Mma	>32	>32	89	0	0		
AXO	Mab	>64	>64	100	0	0	NA	
	Mma	>64	>64	89	0	0		
AUG2	Mab	>64/32	>64/32	100	0	0	NA	
	Mma	>64/32	>64/32	89	0	0		
BDQ	Mab	0.12	0.5					
	Mma	0.12	0.5					
CFZ	Mab	0.25	0.5					
	Mma	0.25	0.5					
DLM	Mab	>0.5	>0.5					
	Mma	>0.5	>0.5					
CPS	Mab	>20	>20					
	Mma	>20	>20					

^*a*^ AMI, amikacin; AUG2, amoxicillin/clavulanic acid 2:1 ratio; AXO, ceftriaxone; BDQ, bedaquiline; CFZ, clofazimine; CIP, ciprofloxacin; CLA, clarithromycin; CPM, capreomycin; DLM, delamanid; DOX, doxycycline; FEP, cefepime; FOX, cefoxitin; IMI, imipenem; LZD, linezolid; MIN, minocycline; MXF, moxifloxacin; SXT, timethoprim/sulfamethoxazole; TGC, tigecycline; TOB, tobramycin.

^*b*^ I, intermediate; R, resistant; S, susceptible.

^*c*^ Pearson *χ*^2^ test.

^*d*^ *χ*^2^ test for continuity correction.

^*e*^ Fisher's exact probability test.

^*f*^ Fisher-Freeman-Halton test.

^*g*^ "–", does not contain the intermediate type.

Among these isolates, Mab exhibited the lowest resistance rates to tigecycline (0%) and amikacin (2%), followed by clarithromycin (27%), linezolid (50%), and cefoxitin (71%). Resistance rates to the other drugs exceeded 96%. For Mma, the lowest resistance rates were observed for amikacin (1%) and tigecycline (6%), followed by clarithromycin (17%), linezolid (44%), cefoxitin (63%), and minocycline (74%). Similarly, resistance rates to the remaining drugs exceeded 92%. Significant differences in clarithromycin (*P* = 0.001) and minocycline (*P* < 0.001) resistance rates were observed between Mab and Mma. *In vitro* tests demonstrated strong anti-microbial activity of bedaquiline and clofazimine against clinical MABC isolates, with an MIC90 value of 0.5 µg/mL. However, all clinical MABC isolates exhibited minimum inhibitory concentration (MIC) values at or above the detection threshold for delamanid and quinolones.

### Correlation between DST and MABC incubation time

We selected four drugs, namely, clarithromycin, tigecycline, linezolid, and amikacin, with sensitivity rates exceeding 20% in clinical MABC isolates, to investigate changes in drug susceptibility over a defined period (from days 4 to 14). Visual representations of the temporal shifts in drug susceptibility can be found in Table S3. Notably, a significant difference was observed in the proportion of clarithromycin-induced changes in drug susceptibility between Mab and Mma strains (*P* < 0.001) (Fig. S2).

### DST in different phenotypes of MABC

We conducted *in vitro* drug susceptibility testing on 193 clinical isolates of MABC, including 97 rough-type strains and 96 smooth-type strains. The results of *in vitro* drug susceptibility testing for various MABC phenotypes, involving 15 commonly used drugs, can be found in Table 3.

**TABLE 3:** MICs of rough and smooth types of *Mycobacterium abscessus* complex

Drugs^*a*^	Colony	MIC50 (µg/mL)	MIC90 (µg/mL)	R^*b*^ (*n*)	I^*b*^ (*n*)	S^*b*^ (*n*)	*χ* ^2^	*P*
	Morphology							
TGC	Rough	0.5	2	3	–^*g*^	93	0	0.991^*c*^
	Smooth	0.12	2	2	–	95		
AMI	Rough	8	16	2	0	94	3.02	0.432^*f*^
	Smooth	8	16	1	3	93		
CLA	Rough	0.5	>16	22	6	68	0.174	0.917^*c*^
	Smooth	0.5	>16	21	5	71		
LZD	Rough	8	32	22	26	48	54.381	0.000^*c*^
	Smooth	32	>32	71	17	9		
DOX	Rough	>16	>16	87	7	2	8.153	0.006^*f*^
	Smooth	>16	>16	96	0	1		
SXT	Rough	>8/152	>8/152	96	–	0	NA	0.497^*e*^
	Smooth	>8/152	>8/152	95	–	2		
MIN	Rough	>8	>8	81	5	10	4.312	0.116^*c*^
	Smooth	>8	>8	87	7	3		
FOX	Rough	128	>128	80	15	1	20.736	0.000^*f*^
	Smooth	64	>128	49	48	0		
MXF	Rough	>8	>8	90	5	1	1.541	0.637^*f*^
	Smooth	>8	>8	94	2	1		
TOB	Rough	16	>16	94	2	0	0	1.000^*d*^
	Smooth	16	>16	94	3	0		
IMI	Rough	>64	>64	96	0	0	1.333	0.248^*d*^
	Smooth	64	>64	94	3	0		
CIP	Rough	>4	>4	96	0	0	NA	
	Smooth	>4	>4	97	0	0		
FEP	Rough	>32	>32	96	0	0	NA	
	Smooth	>32	>32	97	0	0		
AXO	Rough	>64	>64	96	0	0	NA	
	Smooth	>64	>64	97	0	0		
Aug-02	Rough	>64/32	>64/32	96	0	0	NA	
	Smooth	>64/32	>64/32	97	0	0		
BDQ	Rough	0.12	0.25					
	Smooth	0.12	0.25					
CFZ	Rough	0.25	0.5					
	Smooth	0.25	0.5					
DLM	Rough	>0.5	>0.5					
	Smooth	>0.5	>0.5					
CPS	Rough	>20	>20					
	Smooth	>20	>20					

^*a*^ AMI, amikacin; AUG2, amoxicillin/clavulanic acid 2:1 ratio; AXO, ceftriaxone; BDQ, bedaquiline; CFZ, clofazimine; CIP, ciprofloxacin; CLA, clarithromycin; CPM, capreomycin; DLM, delamanid; DOX, doxycycline; FEP, cefepime; FOX, cefoxitin; IMI, imipenem; LZD, linezolid; MIN, minocycline; MXF, moxifloxacin; SXT, timethoprim/sulfamethoxazole; TGC, tigecycline; TOB, tobramycin.

^*b*^ I, intermediate; R, resistant; S, susceptible.

^*c*^ Pearson *χ*^2^ test.

^*d*^ *χ*^2^ test for continuity correction.

^*e*^ Fisher's exact probability test.

^*f*^ Fisher-Freeman-Halton test.

^*g*^ "–", does not contain the intermediate type.

Among rough-type MABC strains, the lowest resistance rates were observed for amikacin and tigecycline, both at 2% (2 of 96). Following these were clarithromycin and linezolid, with resistance rates of 23% (22 of 96). However, the resistance rates for the remaining drugs exceeded 90%. In smooth-type MABC strains, the lowest resistance rates were found for amikacin and tigecycline, each at 1% (1 of 97). The next highest resistance rates were observed for clarithromycin, cefoxitin, and linezolid, with resistance rates of 22% (21 of 97), 51% (49 of 97), and 73% (71 of 97), respectively. For the other drugs, resistance rates also exceeded 90%. A comparison between rough-type and smooth-type MABC clinical isolates revealed significant differences in drug resistance, particularly for linezolid (*P* < 0.001), cefoxitin (*P* < 0.001), and doxycycline (*P* = 0.006).

### *rrl* gene mutations and clarithromycin DST

In all Mab strains, 11 mutation sites and 1 deletion site were identified, while all Mma strains exhibited 10 mutation sites (Table S4). No mutations were detected within the two Mbo strains. Notably, when the MIC of MABC to clarithromycin exceeded 16 µg/mL, 24% (5 of 21) of Mab strains and 57% (8 of 14) of Mma strains exhibited a mutation at position 2270 (*Escherichia coli* numbering 2058) of the *rrl* gene, resulting in the substitution of the original adenine (A) base with guanine (G). This mutation was not observed when the MIC of MABC to clarithromycin was ≤16 µg/mL.

### *erm(41*) gene mutations and clarithromycin DST

Distinct mutation patterns within the *erm(41*) gene were observed among Mab strains with varying clarithromycin susceptibility, as shown in Table S5. Among the strains sensitive to clarithromycin, 65% (17 of 26) exhibited a mutation at position 28 of the *erm(41*) gene, resulting in the substitution of the original thymine (T) base with cytosine (C). In contrast, among those with acquired resistance (1 of 36) and inherent resistance (1 of 27) to clarithromycin, only one strain in each group exhibited a C base mutation at position 28 of the *erm(41*) gene. Furthermore, a significant difference in the mutation at position 255 was observed between Mab strains susceptible to clarithromycin and those resistant to it (*χ^2^* = 14.584, *P* = 0.001). It is worth noting that this specific mutation is synonymous, and its precise implications remain unclear. All 89 strains of Mma exhibited a consistent deletion in the *erm(41*) gene.

### *rrs* gene mutations and amikacin DST

For Mab strains, three consistent mutation sites were found, while Mma strains exhibited seven mutation sites (Table S6). In contrast, the two Mbo strains showed no mutations. Notably, 67% (two of three) amikacin-resistant strains displayed a mutation at position 1375 (*Escherichia coli* numbering 1408) of the *rrs* gene, resulting in the substitution of the original A base with G. Interestingly, this mutation was not observed in strains susceptible or with intermediate susceptibility to amikacin. Importantly, when the MIC of amikacin was ≤1 µg/mL, no base mutations were detected.

## DISCUSSION

Our investigation reveals that Mab is the most prevalent, followed by Mma, and the least common Mbo within MABC. Tigecycline and amikacin demonstrated the highest sensitivity against MABC, followed by clarithromycin and linezolid. Bedaquiline and clofazimine demonstrated notable anti-bacterial activity against MABC, whereas doramapimod and capreomycin were less effective. Mutations in *erm(41*) at position 28 and *rrl* genes at position 2270 (*Escherichia coli* numbering 2058) indicated resistance to clarithromycin, while the *rrs* gene mutation at position 1375 (*Escherichia coli* numbering 1408) was associated with amikacin resistance.

In our study, the distribution of smooth and rough strains was almost equal. Rüger et al. analyzed 50 MABC isolates from 34 patients, finding 50% rough, 38% smooth, and 12% mixed morphologies (20). Their results are consistent with the overall morphological distribution observed in our study. However, they noted significant differences at the subspecies level: 88% of Mab isolates were rough, while 85% of Mbo isolates were smooth (20). In a separate study by Kim et al., 54.1% of Mab isolates were rough; 27% were smooth; and 7% were mixed. For Mma, 71.7% were rough and 28.3% were smooth, while Mbo only had a single smooth isolate (21). In our study, we did not observe noticeable variations in phenotype distribution at the subspecies level.

The distribution of MABC subspecies varies across different regions of China, highlighting the importance of subspecies-level identification for clinical management. Resistance rates also differ between subspecies. A comprehensive study in Beijing found that Mab and Mma constituted 73.8% and 26.2% of MABC isolates, respectively (22). In Shanghai, 75.9% of isolates were Mab, while 24.1% were Mma in a study of 162 isolates (23). Another study in Shanghai reported 74.4% Mab and 25.6% Mma among 43 MABC isolates, while another study in Shanghai found that Mab, Mbo, and Mma accounted for 75.2%, 14.7%, and 10.1%, respectively, in 129 MABC isolates (24). These findings differ from those of our study in southern Guangdong, which provides new data on MABC subspecies distribution in this specific region.

The pathogenicity of NTM is complex and depends on a range of factors, including both environmental determinants and host-related characteristics, with particular emphasis on the latter (25). Our study introduces new insights, suggesting limited clinical relevance of MABC isolates in patients treated for tuberculosis for over 2 months in southern China. Whether prolonged colonization induces pulmonary damage warrants further exploration.

Accurate subspecies identification and comprehensive mycobacterial analysis are critical for predicting treatment outcomes in MABC pulmonary cases (12). Studies in China have reported modest resistance rates for MABC against tigecycline (12.4%) and amikacin (3.9%), along with partial resistance to cefoxitin (39.5%) and imipenem (40.3%), which align with our findings (24). Another study from Southwest China also found that linezolid, amikacin, and cefoxitin were the most effective agents against MABC (3). A comparison of drug susceptibility patterns among the major MABC species, Mab and Mma, indicated a significant difference in clarithromycin-inducible resistance rates (65.67% vs 2.22%) between the two species (4). Comparing Mab, Mbo, and Mma, we noted higher resistance in Mab and Mbo to amikacin and imipenem, while Mab shows lower resistance to tigecycline compared to Mma and Mbo (24). In our study, Mab displayed significantly higher resistance to clarithromycin and minocycline than Mma. These results all indicate that Mab isolates have a higher drug-resistance rate compared to Mma isolates. However, variations in resistance rates suggest potential geographic influences. Regional differences in drug resistance may be related to factors such as the evolution of local strains and the extent of antibiotic use. In regions with high resistance rates, such as China, anti-microbial susceptibility testing should be conducted before antibiotic treatment. Additionally, limiting the use of unnecessary aminoglycosides and optimizing treatment regimens may help slow the further development of resistance. The importance of combination therapy should also be emphasized to ensure effective treatment. Notably, one patient provided sputum samples with a 3 month interval, both identifying Mab. Though drug susceptibility profiles were consistent, slight differences in MIC (e.g., amikacin 16 µg/mL in one sample and 4 µg/mL in the other) might be attributed to variations in sputum origins or experimental and interpretational factors.

The relationship between colony morphology and drug susceptibility requires further investigation due to inconsistent results and regional variations. Rüger et al. found no significant impact of colony morphology on susceptibility to primary antibiotics such as clarithromycin, amikacin, and cefoxitin (20). Koh et al. observed a higher prevalence of smooth colony morphology in pre-treatment isolates (12). In Beijing, clarithromycin resistance rates were higher in smooth-type isolates compared to rough-type isolates (26). In our study, no significant differences were found in clarithromycin and amikacin resistance between different phenotypes. However, cefoxitin resistance was significantly higher in rough-type Mab, while smooth-type Mab showed notably higher resistance rates to linezolid and doxycycline. One potential explanation for these inconsistencies may be methodological differences, such as variations in experimental protocols, the types of media used, or the specific assays employed to assess drug resistance. Furthermore, it is important to consider anti-microbial agents may interact with bacterial cells in ways that are not directly influenced by colony morphology. Future research, employing standardized methodologies and larger sample sizes, would be valuable for clarifying these relationships.

Genotype-guided therapy and the role of genetic mutations [*erm(41*), *rrl*, and *rrs*] in clarithromycin and amikacin resistance are crucial for treatment. Shallom et al. (27) found mutations at A2058G (13/15), A2058C (3/15), and A2059G (1/15) positions in the *rrl* gene of clarithromycin-resistant MABC strains, which were consistent with Bastian et al.’s report (28). In a study of 194 clinical MABC isolates from Shanghai, 13 (6.7%) exhibited clarithromycin resistance; 7 of these resistant strains had mutations at *rrl* gene positions 2270 and 2271, in agreement with our findings (29). The use of efflux pump inhibitors significantly reduced MIC of clarithromycin in non-susceptible isolates, especially in those with intrinsic resistance and lacking *rrl* 2270/2271 mutations (29). However, Carneiro et al. reported different findings, suggesting no clear connection between *erm(41*) and *rrl* gene mutations and clarithromycin resistance in the MABC (30). In Beijing, China, a comprehensive study found that among clinical MABC isolates (7 of 75), 9.3% displayed amikacin resistance, all indicating *rrs* gene mutations. Specifically, six strains had A1408G mutations, and one strain had an A1409G mutation (31). Similarly, a study from Shanghai confirmed the prevalence of the *rrs* gene A1408G mutation in amikacin-resistant strains (80%, four of five) (32). In a Korean study, *rrs* gene mutations were widespread in amikacin-resistant isolates across the *Mycobacterium avium* complex and MABC, with the A1496G mutation being particularly frequent (33).

This study was conducted at a single hospital and thus has certain regional limitations. In the future, this limitation could be mitigated by establishing collaborative partnerships with hospitals of varying levels (e.g., primary care hospitals, specialty hospitals, and large general hospitals) to conduct joint research projects.

This study supports established associations between gene mutations and resistance to clarithromycin and amikacin. The identification of unreported mutations underscores the need for further investigation to fully understand their implications. Our findings contribute to the expanding knowledge of MABC subspecies, susceptibility patterns, and their clinical relevance.

## MATERIALS AND METHODS

### Sample collection

In this retrospective study, a total of 196 MABC isolates were obtained from patients in southern China and submitted to the laboratory at Guangzhou Chest Hospital. These isolates were derived from 195 non-CF patients. The MABC isolates were derived from both pulmonary (*n* = 193) and extrapulmonary (*n* = 3) specimens. The predominant sample type was sputum (84.2%, 165 of 196), followed by bronchoalveolar lavage (14.3%, 28 of 196), with a smaller number originating from pus (2 of 196) or urine (1 of 196).

### Sample processing and culturing

All samples were cultured on Lowenstein-Jensen (LJ) medium. Single colonies of MABC were carefully selected from the LJ culture medium (Baso Diagnostics Inc., Zhuhai, China), then inoculated into 7H9 medium supplemented with 0.05% Tween-80 and 10% oleic acid-albumin-dextrose-catalase (OADC). The cultures were then incubated at 37°C in a shaking incubator.

### Identification

All MABC isolates were previously identified using the Capital Bio Mycobacterial Species Identification Array Kit (Beijing Boao Crystal Code Biotechnology Co., Ltd., Beijing, China) through advanced biochip technology. This kit is designed to distinguish both the *M. tuberculosis* complex and 16 NTM species, including but not limited to *Mycobacterium fortuitum*, *Mycobacterium avium*, *Mycobacterium chelonae*, *Mycobacterium kansasii*, *Mycobacterium ulcerans*, *Mycobacterium intracellulare*, *Mycobacterium aurum*, *Mycobacterium flavescens*, *Mycobacterium gordonae*, *Mycobacterium scrofulaceum*, *Mycobacterium terrae*, *Mycobacterium phlei*, *Mycobacterium avium*, *Mycobacterium smegmatis*, and *Mycobacterium buforanae*. Notably, this identification process is accomplished with a single comprehensive test.

### Drug susceptibility testing

DST was carried out concurrently with genomic DNA extraction using a prospective approach. The testing adhered to the Clinical and Laboratory Standards Institute standard M24 guidelines. MIC determination was conducted by broth microdilution, employing Sensititre RAPMYCO plates (Thermo Fisher Scientific, Massachusetts, USA), encompassing a comprehensive panel of 19 antibiotics. This panel included 15 common antibiotics (amikacin, moxifloxacin, linezolid, ceftriaxone, cefepime, cefoxitin, tigecycline, tobramycin, minocycline, doxycycline, ciprofloxacin, amoxicillin/clavulanic acid 2:1 ratio, trimethoprim/sulfamethoxazole, imipenem, and clarithromycin) alongside four anti-tuberculosis drugs (bedaquiline, clofazimine, delamanid, and capreomycin). Among the 196 MABC strains, 193 strains successfully completed DST, while 3 strains were excluded due to contamination issues. The antibiotic susceptibility drug concentration range and MIC breakpoint values are detailed in Table S1. All isolates were cultivated at 37°C for 3 to 5 days for each drug, and the MIC was determined as the lowest drug concentration that inhibited visible growth. Plates were further incubated at 37°C for up to 14 days to detect inducible clarithromycin resistance. Each MIC determination was performed in duplicate and repeated a third time if a notable variation in phenotype was observed (e.g., a MIC discrepancy of more than one dilution).

### DNA isolation and whole-genome sequencing

MABC isolates were cultured on LJ medium (Baso Diagnostics Inc.). Scraped colonies were suspended in 0.5 mL of normal saline utilizing a disposable inoculating loop and heat-inactivated at 95°C for 30 minutes. Genomic DNA was extracted utilizing the TIANamp Bacteria DNA Kit (Tiangen Biotech Co., Ltd., Beijing, China), in accordance with the manufacturer’s provided guidelines. DNA libraries were prepared using the TIANSeq DirectFast DNA Library Prep Kit (Illumina, Cambridge, UK), following the manufacturer’s instructions. The concentration and length distribution of the libraries were assessed using the Qubit Fluorometer (version 3.0, Thermo Fisher Scientific) and the Agilent 2100 Bioanalyzer (Agilent, California, USA), respectively. Whole-genome sequencing (WGS) libraries were generated from the extracted genomic DNA using a modified protocol of the Illumina Nextera library kit. These libraries were subjected to a 2 × 150 bp paired-end sequencing on the Illumina NovaSeq platform (Illumina). A total of 196 MABC isolates were sequenced as part of this study.

### WGS analysis

The reference genome sequences of Mab (strain: American Type Culture Collection 19977, ASM6918v1), Mma (strain: Japan Collection of Microorganisms 15300, ASM49726v2), and Mbo (strain: BD, ASM360971v1) were obtained from the National Center for Biotechnology Information, selected in accordance with the type strains listed in the LPSN database. The MABC FASTQ files were analyzed using the Burrow-Wheeler Aligner (version 0.7.13). Variant calling was performed with Bcftools (version 1.7), applying a minimum coverage threshold of 10×. For the construction of a maximum likelihood tree, the PhyML program within IQ-Tree (version 2.0.7) was employed. In addition, SNPs were only at positions consistently shared across all samples. SNPs were considered only if they possessed a coverage of at least 5×, including a minimum of 1× coverage in each direction. Indel-related SNPs within 12 bp of each other were excluded from the analysis. Positions featuring ambiguous base calls were also omitted. To assess genetic relatedness, a distance matrix was generated, and a minimum spanning tree was constructed through permutation resampling, involving 1,000 replicates.

### Statistics analysis

Statistical analysis was conducted using SPSS (version 25.0). Count data were presented as frequency (percentage), and comparisons among multiple groups were performed using the Fisher-Freeman-Halton test. The significance threshold for the test was set at *P* = 0.05, with results deemed statistically significant for *P* values less than 0.05.

## SUPPLEMENTAL MATERIAL

Supplemental figures and tables spectrum.04103-23-s0001.docx Fig. S1 to S2; Tables S1 to S6.

ASM does not own the copyrights to Supplemental Material that may be linked to, or accessed through, an article. The authors have granted ASM a non-exclusive, world-wide license to publish the Supplemental Material files. Please contact the corresponding author directly for reuse.
