# Supplementary material for: Subspecies distribution and drug-resistance characteristics of Mycobacterium abscessus complex clinical isolates in South China
Source: Microbiol Spectr. 2025 Mar 19;13(5):e04103-23. doi: 10.1128/spectrum.04103-23 (PMC12054122; doi:10.1128/spectrum.04103-23)
Supplement: Supplemental figures and tables — Fig. S1 to S2; Tables S1 to S6. [file spectrum.04103-23-s0001.docx]

Subspecies distribution and drug resistance characteristics of *Mycobacterium abscessus* complex clinical isolates in south China

**Figure 1: Phylogenetic tree of clinical isolates of *Mycobacterium abscessus* complex**

Maximum likelihood phylogenetic tree of 196 M. abscessus isolates generated by IQ-TREE and displayed with iToL and corresponding subspecies, phenotypic groups, annotated for drug resistance phenotypes (Tigecycline, Amikacin and Clarithromycin MIC day 3 & 14).

**Figure 2: Relationship between time of MABC isolation, duration of anti-tuberculosis treatment, and progression of pulmonary disease.**

MABC: *Mycobacterium abscessu*s complex; Mab: *Mycobacterium abscessus* subsp. *abscessus*; Mma: *Mycobacterium abscessus* subsp. *massiliense;* Mma: *Mycobacterium abscessus* subsp. *bolletii*.

**SUPPLEMENTARY FIGURE LEGENDS**

**Supplementary Figure 1: Distribution of clinical isolates of *Mycobacterium abscessus* complex in Guangdong Region.** Different colors represent the collection quantities of clinical isolates of *Mycobacterium abscessus* complex in various regions of Guangdong. 16 patients originated from other provinces, including 3 from Fujian, 3 from Sichuan, 2 from Guangxi, 2 from Hainan, 2 from Jiangxi, 1 from Hunan, 1 from Hubei, 1 from Guizhou, and 1 from Gansu. The geographical origin of the remaining 55 patients remained unknown.

**Supplementary Figure 2: Changes in drug susceptibility of clarithromycin.** A1: Illustrates the changes in clarithromycin susceptibility over time for *Mycobacterium abscessus* subsp. abscessus. A2: Illustrates the changes in clarithromycin susceptibility over time for *Mycobacterium abscessus* subsp. *massiliense.*

**Supplementary Figure 1**

**
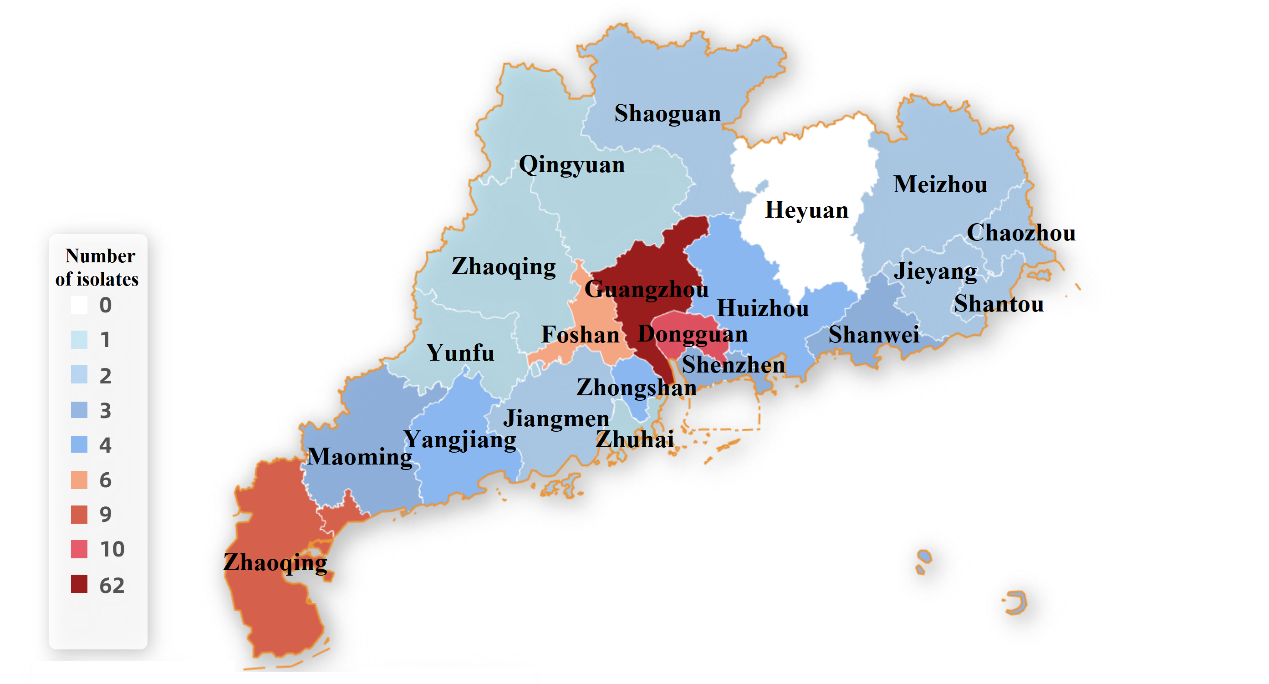
**

**Supplementary Figure 2:**


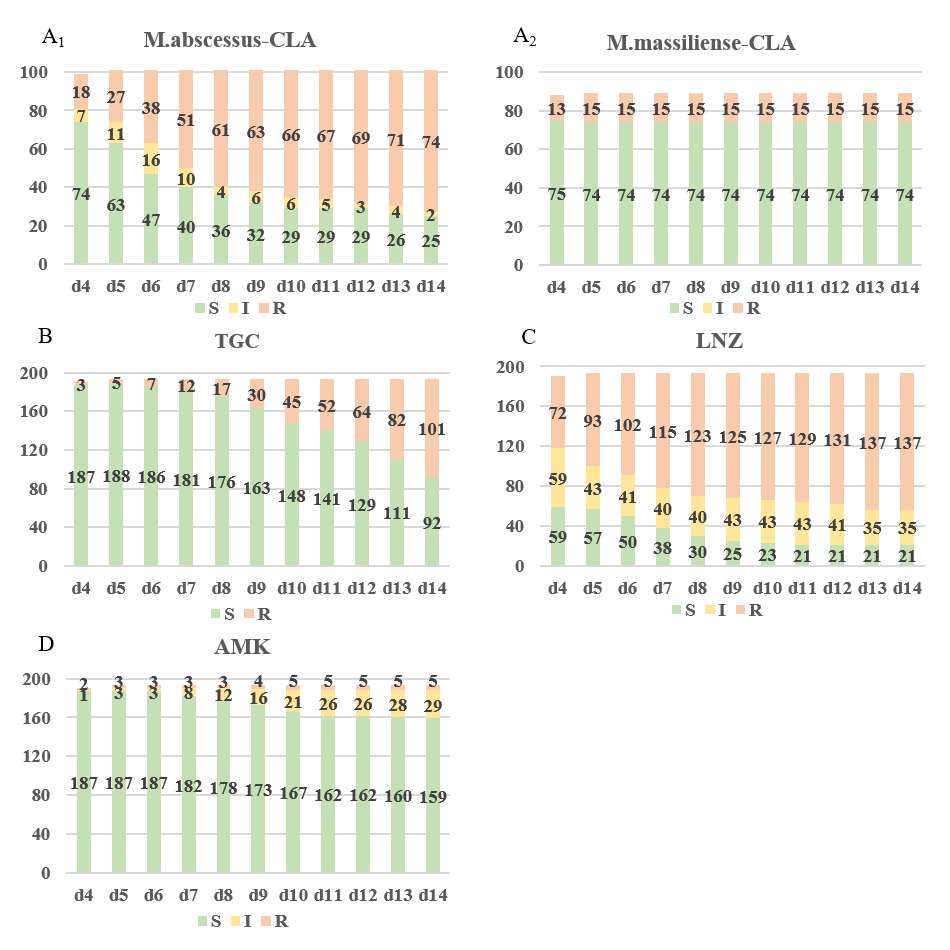


**Supplementary Table**

**Supplementary Table 1 Antibiotic susceptibility drug concentration range and MIC breakpoint values**

| Drugs* | MIC range |  | Breakpoint(µg/mL) | | |
| --- | --- | --- | --- | --- | --- |
|  | (µg/mL) |  | Susceptible | Intermediate | Resistant |
| Aminoglycosides |  |  |  |  |  |
| AMI | 1-64 |  | ≤16 | 32 | ≥64 |
| TOB | 1-16 |  | ≤2 | 4 | ≥8 |
| Cephalosporins |  |  |  |  |  |
| FOX | 4-128 |  | ≤16 | 32–64 | ≥128 |
| FEP | 1-32 |  | ≤8 | 16 | ≥32 |
| AXO | 4-64 |  | ≤16 | 32 | ≥64 |
| Carbapenems |  |  |  |  |  |
| IMI | 2-64 |  | ≤4 | 8–16 | ≥32 |
| Fluoroquinolones |  |  |  |  |  |
| CIP | 0.12-4 |  | ≤1 | 2 | ≥4 |
| MXF | 0.25-8 |  | ≤1 | 2 | ≥4 |
| Folate pathway inhibitors |  |  |  |  |  |
| SXT | 0.25/4.75-8/152 |  | ≤2/38 | – | ≥4/76 |
| Tetracyclines |  |  |  |  |  |
| TGC | 0.015-4 |  | <4 | – | ≥4 |
| MIN | 1-8 |  | ≤1 | 2–4 | ≥8 |
| DOX | 0.12-16 |  | ≤1 | 2–4 | ≥8 |
| Macrolides |  |  |  |  |  |
| CLA | 0.06-16 |  | ≤2 | 4 | ≥8 |
| Oxazolidinones |  |  |  |  |  |
| LZD | 1-32 |  | ≤8 | 16 | ≥32 |
| β-lactams |  |  |  |  |  |
| AUG2 | 2/1-64/32 |  | ≤16/8 | 32/16 | ≥64/32 |
| Antituberculosis |  |  |  |  |  |
| BDQ | 0.06-8 |  |  |  |  |
| CFZ | 0.06-8 |  |  |  |  |
| DLM | 0.008-0.5 |  |  |  |  |
| CPS | 0.15-20 |  |  |  |  |

*AMI: Amikacin, TOB: Tobramycin, FOX: Cefoxitin, FEP: Cefepime, AXO: Ceftriaxone, IMI: Imipenem, CIP: Ciprofloxacin, MXF: Moxifloxacin, SXT: Timethoprim/sulfamethoxazole, TGC: Tigecycline, MIN: Minocycline, DOX: Doxycycline, CLA: Clarithromycin, LZD: Linezolid, AUG2: Amoxicillin/clavulanic acid 2:1 ratio, IMI: Imipenem, BDQ: Bedaquiline, CFZ: Clofazimine, DLM: Delamanid, and CPM: Capreomycin

**Supplementary Table 2: Distribution of colony morphology of different subspecies of *Mycobacterium abscessus* complex (n)**

|  | Smooth | Rough | N | *χ^2^* | *p* |
| --- | --- | --- | --- | --- | --- |
| Mab | 49 | 54 | 103 | 0.171 | 0.679 |
| Mma | 45 | 44 | 89 |  |  |
| Mbo | 2 | 0 | 2 |  |  |
| Mab and Mma | 2 | 0 | 2 |  |  |
| Total | 98 | 98 | 196 |  |  |

Mab: *Mycobacterium abscessus* subsp. *abscessus*; Mma: *Mycobacterium abscessus* subsp. *massiliense*; Mbo: *Mycobacterium abscessus* subsp. *bolletii*.

**Supplementary Table 3: Comparison of the proportion of drug susceptibility result changes with prolonged culture time in *Mycobacterium abscessus* subspecies *abscessus* and *Mycobacterium* *massiliense* subspecies *massiliense***

| Drug* | Mab | | Mma | | *χ*^2^ | *p* |
| --- | --- | --- | --- | --- | --- | --- |
|  | day4S+day14S^a^ | day4S+day14R^b^ | day4S+day14S^a^ | day4S+day14R^b^ |  |  |
| CLA | 26 | 37 | 75 | 0 | 60.184 | 0.000 |
| TGC | 50 | 51 | 41 | 44 | 0.030 | 0.884 |
| LNZ | 20 | 9 | 20 | 9 | 0.041 | 0.839 |
| AMI | 98 | 0 | 86 | 1 | - | 0.470 |

Mab: *Mycobacterium abscessus* subsp. *abscessus*; Mma: *Mycobacterium abscessus* subsp. *massiliense*; *AMI: Amikacin, CLA: Clarithromycin, TGC: Tigecycline, LZD: Linezolid; ^a^day4S+day14S:When reading the antibiotic susceptibility results on the 4th day, the result was "S" (Susceptible); and when reading the antibiotic susceptibility results on the 14th day, it was still "S"; ^b^day4S+day14R: When reading the antibiotic susceptibility results on the 4th day, the result was "S"; however, when reading the antibiotic susceptibility results on the 14th day, it was "R" (Resistant).

**Supplementary Table 4: *rrl* Gene mutations and Clarythromycin MIC for MABC**

| MIC | Mab | | | | |  | Mma | | | | | |
| --- | --- | --- | --- | --- | --- | --- | --- | --- | --- | --- | --- | --- |
| (µg/ml) | N | G17A | A180G | A2270G | C3042T |  | N | C111T | G400A | T1568C | C1613A | A2270G |
| ≤0.06 | 3 | 0 | 0 | 0 | 0 |  | 27^c^ | 12 | 1 | 1 | 0 | 0 |
| 0.12 | 11 | 0 | 0 | 0 | 2 |  | 21 | 8 | 2 | 0 | 1 | 0 |
| 0.25 | 7 | 1 | 0 | 0 | 0 |  | 17 | 6 | 6 | 1 | 1 | 0 |
| 0.5 | 3 | 0 | 0 | 0 | 0 |  | 6 | 0 | 0 | 0 | 0 | 0 |
| 1 | 2 | 1 | 0 | 0 | 1 |  | 3 | 0 | 0 | 0 | 0 | 0 |
| 4 | 11 | 0 | 0 | 0 | 0 |  | 0 | 0 | 0 | 0 | 0 | 0 |
| 8 | 5 | 0 | 1 | 0 | 3 |  | 1^d^ | 0 | 0 | 0 | 0 | 0 |
| 16 | 1 | 0 | 0 | 0 | 0 |  | 0 | 0 | 0 | 0 | 0 | 0 |
| >16 | 21^a^ | 0 | 1 | 5 | 1 |  | 14 | 0 | 1 | 0 | 0 | 8 |
| Induced resistance | 36^b^ | 0 | 1 | 0 | 3 |  | 0 | 0 | 0 | 0 | 0 | 0 |
| Total | 100 | 2 | 3 | 5 | 10 |  | 89 | 26 | 10 | 2 | 2 | 8 |
| Mab: *Mycobacterium abscessus* subsp. *abscessus*; Mma: *Mycobacterium abscessus* subsp. *massiliense*; ^a^ Three strains of bacteria had the mutations G1610A, T2343C, and T2823C, respectively; ^b^ Four strains of bacteria had the mutations T630C, C712T, G2415A, and ACC2978ACCC, respectively; ^c^ Another strain had a C229T mutation; ^d^ Another strain had an A2271G mutation. | | | | | | | | | | | | |

**Supplementary Table 5: Analysis of *erm(41)* gene variations in different in vitro clarithromycin susceptibility results among subtypes of *Mycobacterium abscessus* complex**

| Mutation Site | Mutation Type | Mutation Proportion[n(%)] | | | *χ^2^*^a^ | *p* |
| --- | --- | --- | --- | --- | --- | --- |
|  |  | CLA-Induced resistance | CLA-S | CLA-R |  |  |
| 28 | T>C | 1(0.03) | 18(0.72) | 1(0.04) | 32.966 | 0.000 |
| 120 | A>G | 7(0.19) | 0(0.00) | 5(0.19) | 3.744 | 0.053 |
| 158 | G>A | 0(0.00) | 2(0.08) | 0(0.00) | - | 0.164 |
| 159 | T>C | 18(0.50) | 18(0.72) | 14(0.52) | 2.952 | 0.086 |
| 168 | G>C | 8(0.22) | 2(0.08) | 4(0.15) | 1.263 | 0.261 |
| 238 | A>G | 18(0.50) | 18(0.72) | 14(0.52) | 2.952 | 0.086 |
| 255 | G>A | 17(0.47) | 2(0.08) | 12(0.44) | 10.584 | 0.001 |
| 276 | A>T | 0(0.00) | 0(0.00) | 1(0.04) | - | - |
| 279 | G>T | 12(0.33) | 2(0.08) | 12(0.44) | 5.355 | 0.021 |
| 310 | C>T | 0(0.00) | 1(0.04) | 0(0.00) | - | 0.410 |
| 330 | A>C | 20(0.56) | 20(0.80) | 15(0.56) | 3.905 | 0.048 |
| 336 | T>C | 11(0.31) | 2(0.08) | 13(0.48) | 4.716 | 0.030 |
| 419 | C>T | 2(0.06) | 1(0.04) | 3(0.11) | 0.000 | 1.000 |
| 450 | G>A | 0(0.00) | 0(0.00) | 1(0.04) | - | - |

^a^ Performing a chi-square test on the data for sensitivity to clarithromycin and resistance to clarithromycin.

**Supplementary Table 6: Relationship between *rrs* Gene and MIC Values of Amikacin in Mab Subtypes and Mma Subtypes**

| MIC | Mab | |  | Mma | | | | |
| --- | --- | --- | --- | --- | --- | --- | --- | --- |
| (µg/mL) | N | A1375G |  | N | A976G | C977T | T1233C | A1375G |
| ≤1 | 2 | 0 |  | 0 | 0 | 0 | 0 | 0 |
| 2 | 5 | 0 |  | 4 | 1 | 2 | 0 | 0 |
| 4 | 23 | 0 |  | 23 | 8 | 9 | 1 | 0 |
| 8 | 55 | 0 |  | 43 | 10 | 19 | 1 | 0 |
| 16 | 12 | 0 |  | 16 | 2 | 6 | 1 | 0 |
| 32 | 1 | 0 |  | 2 | 0 | 2 | 0 | 0 |
| >64 | 2 | 1 |  | 1 | 0 | 1 | 0 | 1 |
| N | 100 | 1 |  | 89 | 21 | 39 | 3 | 1 |

Mab: *Mycobacterium abscessus* subsp. *abscessus*; Mma: *Mycobacterium abscessus* subsp. *massiliense*;
